# Precise Serial Microregistration Enables Quantitative Microscopy Imaging Tracking of Human Skin Cells In Vivo

**DOI:** 10.3390/cells13131158

**Published:** 2024-07-07

**Authors:** Yunxian Tian, Zhenguo Wu, Harvey Lui, Jianhua Zhao, Sunil Kalia, InSeok Seo, Hao Ou-Yang, Haishan Zeng

**Affiliations:** 1 Imaging Unit, Integrative Oncology Department, BC Cancer Research Centre, Vancouver, BC V5Z 1L3, Canada; 2 Photomedicine Institute, Department of Dermatology and Skin Science, University of British Columbia, Vancouver, BC V5Z 4E8, Canada; 3 Vancouver Coastal Health Research Institute, Vancouver, BC V5Z 1M9, Canada; 4 Johnson and Johnson Consumer Inc., Skillman, NJ 08558, USA

**Keywords:** in vivo microscopy, reflectance confocal microscopy, multiphoton microscopy, two-photon excitation fluorescence, second harmonic generation, in vivo cellular imaging, in vivo cell tracking, human skin, melanin, epidermal, UV radiation, longitudinal study

## Abstract

We developed an automated microregistration method that enables repeated in vivo skin microscopy imaging of the same tissue microlocation and specific cells over a long period of days and weeks with unprecedented precision. Applying this method in conjunction with an in vivo multimodality multiphoton microscope, the behavior of human skin cells such as cell proliferation, melanin upward migration, blood flow dynamics, and epidermal thickness adaptation can be recorded over time, facilitating quantitative cellular dynamics analysis. We demonstrated the usefulness of this method in a skin biology study by successfully monitoring skin cellular responses for a period of two weeks following an acute exposure to ultraviolet light.

## 1. Introduction

Serial analysis of human cellular dynamics in vivo over time can provide unique insights into biological behavior [1,2,3]. Until now, most of the studies have been carried out on biopsy ex vivo samples, which preclude monitoring of the same tissue microlocation and specific cells [4]. These approaches are also time consuming, invasive, destructive, require more study subjects for achieving statistically meaningful results, and lack reliability in inferring cellular or molecular dynamics because data at different time points are from different subjects or different tissue sites of the same subject. Ideally, in order to directly observe the temporal evolution of living processes, the same tissue microlocations, including their specific cells and other structures, should be monitored non-invasively in vivo for an extended period of time and with high spatial resolution. Optical multimodal microscopy imaging, including integrated reflectance confocal and multiphoton microscopy, is a high-resolution label-free technique that enables real-time in vivo live cell imaging without physical tissue dissection [5,6,7], and thus has the potential to do the job. However, imaging cell dynamics over time in human cells in vivo bears tremendous challenges [4,7].

**Figure 1:**
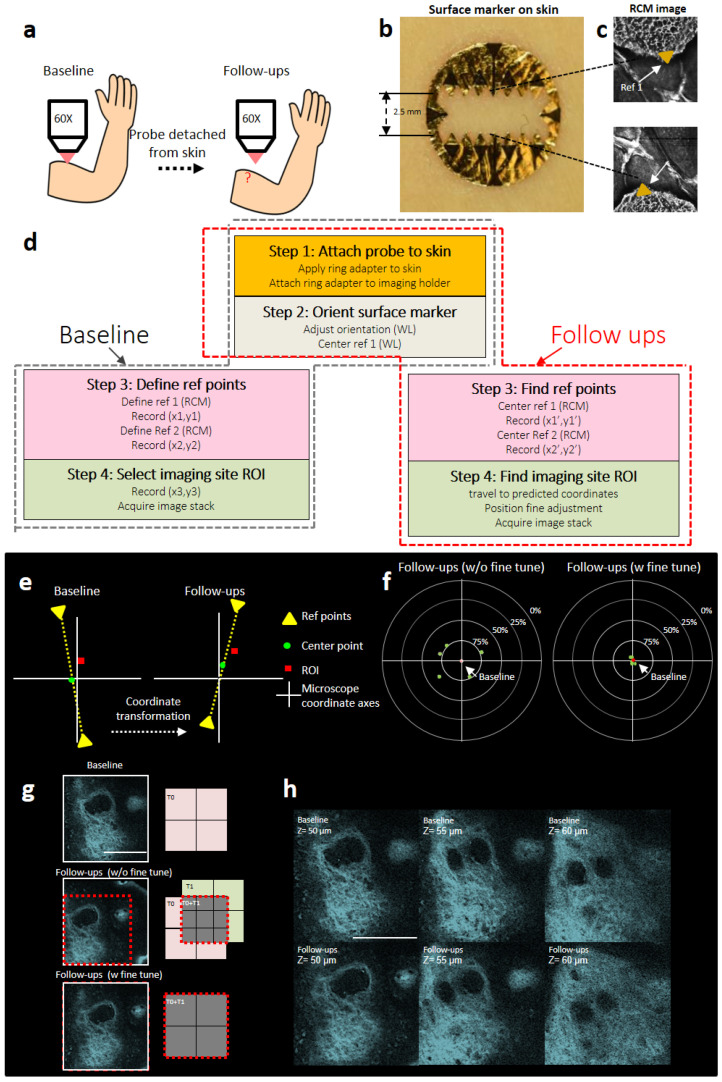
Methodology for precise microregistration that enables quantitative tracking of skin cells in vivo. (**a**) Illustration of the challenge for relocalization when the microscope probe is reattached to the skin at successive imaging sessions; (**b**) example of surface marker design, which has serrated edges that serve as reference markers for microregistration; (**c**) reflectance confocal microscopy (RCM) images of serrated edge tips of the surface marker as references for microregistration; (**d**) procedures for microregistration (for details, see text and Supplemental Figures S1 and S2 and Table S1); (**e**) the location prediction algorithm is based on coordinate transformation calculation, from baseline to follow-up time points; (**f**) the percentage number describes the relative radial distance consistency between the follow-up and the baseline; 100% means follow-up and baseline distance is 0, and 0% means distance is 200 µm. The performance of automatic microregistration without fine-tuning can achieve > 56% of ROI overlap with previous imaging sessions (microregistration accuracy of better than 50 μm in both X and Y directions). With further fine-tuning, the accuracy of registration between imaging sessions is improved and the consistency is close to 100%; (**g**) example of automatic microregistration before (middle panel) and after fine-tuning (bottom panel); shown are in vivo skin dermis second harmonic generation (SHG) images excited by 810 nm fs laser light; (**h**) examples of in vivo dermal SHG images at three different depths taken at two imaging sessions. Scale bar: 100 μm.

The challenge for longitudinal in vivo human skin cell imaging is to pinpoint/relocalize to the same microlocation/specific cells repeatedly and efficiently in multiple imaging sessions over which biological changes occur. Recently, Entenberg et al. [8] proposed a permanent window that was embedded in tissue for high resolution imaging of lung cancer metastasis in a murine model. Jung et al. [9] used a small tattoo that was injected into tissue for longitudinal three-dimensional (3D) imaging of sebaceous glands in response to cryotherapy using a mouse ear model. Although these techniques achieved significant advancement for longitudinal in vivo imaging at the cellular level, they are extremely invasive and limited to animal models. Longitudinal cellular imaging for human subjects in vivo has been limited to a single imaging session, which could last up to several hours in duration at a maximum. Here the imaging head or microscope probe stays at the imaging site during the whole session. For cell tracking over the course of multiple imaging sessions spanning over days or weeks, the imaging head/microscope probe may leave the imaging site between sessions and needs to be relocated to the same microlocation in each session (Figure 1a). In this paper, we present a novel non-invasive method to achieve precise serial microregistration (relocalization to the same microlocation), which allowed us to track the cellular dynamics of human skin responses following an acute exposure to ultraviolet B (UVB) light. Using this method, we demonstrated successful longitudinal monitoring of a number of quantitative assessment parameters including cell proliferation, melanin upward migration, blood flow velocity increasing, and epidermal thickness adaptation over a period of two weeks.

## 2. Materials and Methods

The microregistration technique is based on attaching a temporary “surface marker” landmark, which remains affixed to the skin during the full course of the study without interfering with a volunteer’s daily activities. Seven volunteers (4 male, 3 female), aged 24–55 years with skin types II-IV were included in this study. This study was approved by the Clinical Research Ethics Board of the University of British Columbia (certificate number: H16-00355) and consent was obtained from each volunteer subject before the experiment. 

### 2.1. UVB Illumination 

UVB illumination was first applied to the left upper inner arm of a subject to determine the minimum erythema dose (MED). The right upper inner arm was used for the imaging and cellular response study following a 2 MED single-dose acute UVB exposure. The light source used is a solar simulator based on a 150 watt Xenon arc lamp (L2274, Hamamatsu, Japan) filtered with UG-11 and WG-320 (Schott Optical Company, Duryea, PA, USA) glass filters to provide UV illumination from 280 to 400 nm. The filtered collimated light beam was coupled to an 8 mm diameter quartz liquid light guide. The uniformity of the output light distribution was confirmed using photochromic film. The UVB band (280–320 nm) dominates the biological effects with an irradiance of 0.47 mW/cm^2^. Graded doses of UVB were first applied to the left upper inner arm of each subject to determine the minimum erythema dose (MED) for that individual at that site. This MED phototest was performed by UV exposures at 6–8 skin sites of the left upper inner arm. Each site received a different fluence controlled by time of exposure with geometric increments of 20%. The size of each site of exposure was 1 × 6 mm^2^. Skin responses were graded 24 h after exposure and the minimal erythema doses (MEDs), defined as the lowest UVB fluence that caused minimally perceptible skin redness, were determined by two dermatologists reaching consensus. A single dose of twice the individual MED UVB exposure was administrated to the right upper inner arm of the subject to study the cellular responses by repeated imaging of the same ROIs inside the UV-exposed region for two weeks. 

### 2.2. Multimodal Imaging System

The multimodal imaging system was home-made and the details were described in our previous publication [10]. In the system configuration for this study (Supplementary Figure S3), the output from a tunable (720–950 nm) femtosecond (fs) Ti:Sapphire laser (Chameleon, Coherent Inc., Santa Clara, CA, USA) was scanned by an 8 kHz resonance scanner and a galvanometer scanner over the back aperture of a 60× (NA = 1.0) water-immersion objective, then it reached the skin. The objective lens was mounted on an imaging holder, and its location and focal plane were controlled by a motorized micrometer-actuated 3-axis translation stage. In the detection path, the objective collected two-photon excitation fluorescence (TPF) and second harmonic generation (SHG) signals in the epi-direction and a 665 nm long pass dichroic was placed behind the objective to reflect these imaging signals towards the detectors. The TPF and SHG signals were further separated by a dichroic beam splitter and detected by a pair of photomultiplier tube (PMT) modules to form the TPF and SHG images. A 50/50 beam splitter was used to direct the backscattered and descanned reflectance confocal microscopy (RCM) signal to an avalanche photodiode (APD) module to form the RCM image. The RCM, SHG, and TPF signals were recorded by a frame grabber as synchronized but separate video streams. For that, the TPF, SHG, and RCM images were acquired simultaneously and all modalities were co-registered. The image acquisition speed was about 15 frames/second, allowing real-time visualization of blood flow and other dynamic events. Wide field white light imaging was also integrated into the system. White light LEDs mounted on the imaging holder were turned on to provide illumination. The 665 nm long pass dichroic was rotated 90 degrees to reflect the white light signal to the color CCD camera to acquire reflected white light images of the skin and the surface marker. 

For imaging, the metal ring adapter was affixed onto the subject’s skin using double-sided adhesive film. A coverslip was present between the objective and the skin surface. Water was placed in between the coverslip and the objective lens as well as between the coverslip and the skin. The imaging holder, the objective, the metal ring adapter, and the coverslip together form the microscope probe mentioned earlier. The maximum incident laser power for localization of the surface marker is 10 mW, and for skin imaging it is 40 mW. RCM, TPF, and SHG images parallel to the skin surface were acquired at different depths inside the skin starting at the top of the stratum corneum and ending at 100–150 µm depth, with a step size of 1 μm between images. The measurements were carried out by two excitation wavelengths: 735 nm and 810 nm. The 735 nm light was used to image cells by exciting TPF. The 810 nm light was used to image melanin by exciting TPF and it also induced SHG signals for imaging collagen. The field of view (FOV) for this microscopy imaging was 200 × 200 µm^2^. One horizontal scanned image stack was acquired for each volunteer at each time point. 

### 2.3. Design of Temporary Surface Marker

The temporary surface marker was designed to be about a 10 mm in diameter circular shape with a 2.5 × 8 mm^2^ rectangular opening in the center. The rectangular edge is serrated so that the tips can serve as reference points. The color of the marker was designed in gold and black in order to be easily differentiated from skin tissue under white light imaging and RCM or multiphoton imaging with no restriction of the excitation wavelength. The temporary surface marker sticks on the skin without fading for two weeks, and does not interfere with skin tissue or its function. It was manufactured by Flash Tattoo (https://www.flashtat.com/ (accessed on 1 June 2019)) as a customized order. This tattoo can be used for any skin type. It could be peeled off after the two-week-long study period without leaving any residual marks on the skin. 

### 2.4. Repeated Alignment to the Same Microstructures and Cells within the Region of Interest (ROI) over Time at Different Imaging Sessions

Prior to any imaging, the surface marker was applied onto the subject’s skin (Supplemental Note S1. At baseline imaging, two tips opposite from each other on the surface marker rectangular opening were selected as the navigational reference points. The FOV of white light and RCM were calibrated to be coincident. After the microscope probe was attached onto the skin, the (x, y) coordinates of the two navigational reference points were imaged under RCM mode and recorded by controlling the motorized stage. The coordinates of the region of interest (ROI) were also noted. In follow-up imaging sessions, the (x, y) coordinates of the reference points were again recorded. These (x, y) coordinates were input into a custom MATLAB code to calculate the predicted ROI position. The prediction algorithm of the MATLAB code (Mathworks, Natick, MA, USA) was based on the coordinate transformation equations shown in Supplemental Table S1 and Supplemental Figure S1. When visualizing and tracking the same biological microstructure and specific cells, one can fine-tune the stage until the FOV of imaging the relocated ROI was the same as that of the baseline. The detailed procedure is explained in Supplemental Note S2 and Supplemental Figure S2. 

### 2.5. Method Validation 

Verification of the capability of precise relocalization to revisit the same microlocation day after day can be accomplished by visual identification of microstructures that maintain their morphology between image sessions. The collagen network in dermis as revealed by SHG imaging is easily recognizable from day to day (as shown in Figure 1h) and could serve as such a microsctructure target. 

### 2.6. Image Analysis 

The images were processed using ImageJ (version 1.52p) and MATLAB (version R2020b). 

#### 2.6.1. Blood Flow Velocity Analysis

**Figure 2:**
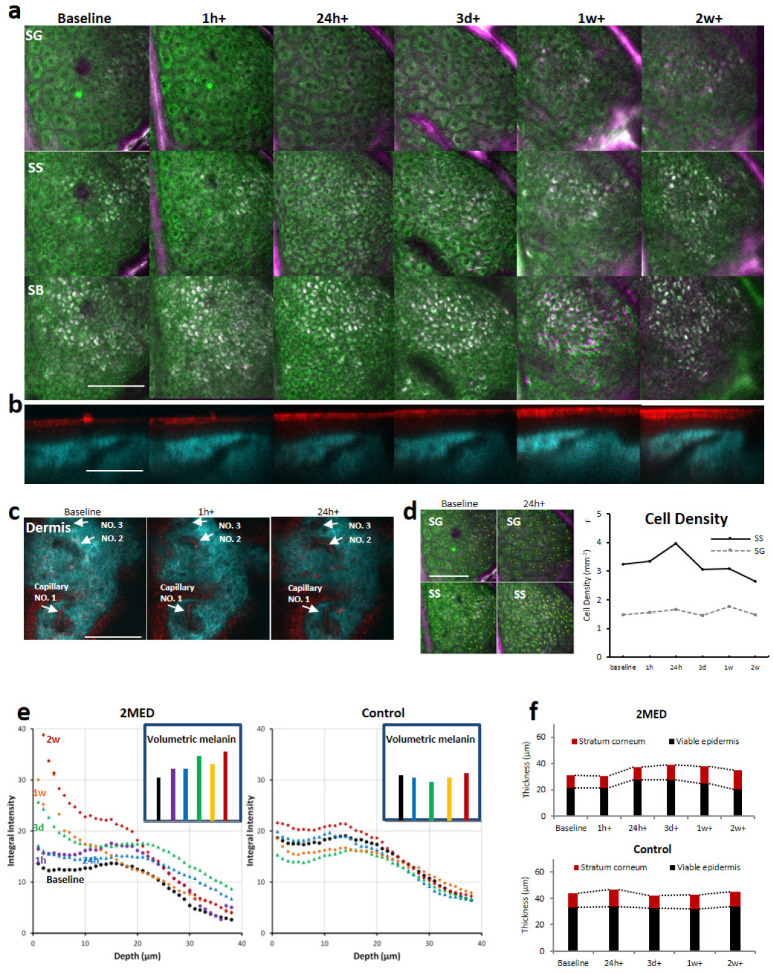
Application of microregistration for in vivo quantitative monitoring of human skin cellular response dynamics to acute UVB exposure. (**a**) Pseudocolor multimodal en face images of different epidermal layers before and after UVB exposure. SG: stratum granulosum; SS: stratum spinosum; SB: stratum basale; magenta for TPF (two-photon fluorescence) image under 810 nm fs laser excitation; green for TPF image under 735 nm excitation. (**b**) Pseudocolor multimodal vertical sectional images of human skin before and after UVB exposure. Cyan for SHG image under 810 nm excitation, red for RCM image at the same excitation wavelength. (**c**) Pseudocolor multimodal en face images of skin dermis before, 1 h, and 24 h after UVB exposure. Cyan for SHG image; red for standard deviation (STD) of the RCM intensity at each pixel calculated from 10 sequential image frames, highlighting blood flow in vessels. (**d**) Left: en face images of SG and SS epidermal layers before (baseline) and 24 h after UVB exposure. Yellow dots indicate the center of keratinocytes. Right: Cell density changes after UVB exposure. Note that cell density in the SS layer increases following UVB exposure and reaches maximum at 24 h, then settles down afterwards. (**e**) Melanin signal at different depth changes with time. Small box: total melanin signal (volumetric integration of presumed melanin signal from all layers) changes over time. The six bars from left to right in the 2MED box represent melanin at baseline, 1 h, 24 h, 3 d, 1 w, and 2 w, respectively. The five bars in the Control box are for melanin at baseline, 24 h, 3 d, 1 w, and 2 w. (**f**) Changes in epidermal thickness after UVB exposure. Note that the thickness of epidermis increases and then decreases between 24 h and 2 weeks with a maximum at 3 days. In contrast, the stratum corneum thickness continues increasing over the 2-week period. The control site shows minimal changes in skin thickness. (h—hour, d—day, w—week.) Scale bar: 100 μm.

The standard deviations (STD) of 10 frames of RCM images were calculated for visualizing capillary blood flow [11]. The RCM STD image (red) merged with the SHG image (cyan) was used to show dermal blood vessels (Figure 2c). 

#### 2.6.2. Presumed Melanin TPF Analysis

The 810 nm excitation TPF signals were used to quantify melanin after removal of the dermal TPF signals from elastin located at the dermal epidermal junction (DEJ) and in the dermis. This was accomplished by utilizing the 810 nm-excited SHG collagen signals. The 810 nm excitation SHG image at each depth in the DEJ and dermis region was segmented using MATLAB. A set of binary masks was created as the pixels inside the collagen area were assigned 0 values, while pixels elsewhere were assigned a value of 1. The dermal TPF signals could be filtered out by multiplying the 810 nm excitation TPF images with corresponding binary masks (Supplemental Figure S5). The TPF intensity was calculated within the remaining epidermal subregion once the dermis region was masked out. The volumetric presumed melanin signal intensity at 810 nm excitation was integrated at depth from the top of the SG layer to the DEJ within the imaging stack.

#### 2.6.3. Skin Layer Thickness Analysis 

The total epidermal thickness, defined as the averaged distance from the skin surface to the dermal-epidermal junction (DEJ), was determined directly from vertical scanned images (as shown in Figure 2). The DEJ was clearly revealed by the SHG signals of collagen fibers under 810 nm fs laser excitation. Ten adjacent vertical section (XZ plane) images were extracted from each 3D stack. We have validated that the same microstructures were imaged at each time point (Figure 2b). The distance from the skin surface to the DEJ at each vertical line of the XZ image was calculated using Matlab. In total, there were about 250 locations within the vertical section image. Distances from all the 256 lines of each of the 10 XZ images were averaged to generate the final mean distance to be used as the total epidermal thickness. The thickness of the stratum corneum (SC) was defined as the distance from the skin surface to the bottom of the SC. The SC layer was clearly visualized by the RCM signal and the TPF signal under 810 nm fs laser excitation. The thickness of the viable epidermis was derived from the difference between the total epidermis thickness and the SC thickness. 

#### 2.6.4. Cell Density

Two epidermal layers, SG and SS, were analyzed at baseline and the 24 h time point. Cells were labeled as yellow dots manually by visually identifying the cell nuclei and cytoplasma. The imaging expert counting the cells was blinded to the time points. The numerical density of granular and spinous keratinocytes (number of cells per en face mm^2^ area) was calculated from the images (highlighted as yellow dots in Figure 2d). Cell density was derived by counting the number of yellow dots and then dividing by the area of the region of interest.

## 3. Results

### 3.1. Serial Registration to the Same Microlocation

The microregistration is realized by attaching a temporary “surface marker” landmark, which remains affixed to the skin during the full course of the study without interfering with a subject’s daily activities. As an example, the surface marker can be configured as a circular shape with a central 2.5 × 6 mm^2^ open window for the region of interest (ROI) to be imaged and tracked. The internal edges of the opening are intentionally serrated with the tips serving as reference points for alignment (Figure 1b). The marker is colored in gold and black (Figure 1b, Supplemental Figure S4b) so that it can be easily distinguished from tissue under white light (WL) (Figure 1b) and reflectance confocal microscopy (RCM) imaging (Figure 1c). The challenge in microregistration is the alignment of the objective with the orientation of the surface marker over successive imaging sessions (Figure 1e). We developed a technique that can automatically correct the rotations and translations of the surface marker relative to the objective at each imaging session (Figure 1d). This was achieved by selecting two opposite sawtooth tip points as fixed reference landmarks for calibration in each imaging session (Supplemental Figure S1). The detailed procedure is shown in Supplemental Figure S2. To assess the performance of the method, we tested the relocation accuracy on human skin in vivo across separate imaging sessions over five days (one imaging session/day). At all times, the same microstructures could be identified across all imaging sessions within a field of view (FOV) of 200 µm x 200 µm. In successive imaging sessions, more than 56% of the ROI area coincided with that of the first baseline imaging session, representing a microregistration accuracy of better than 50 µm in both X and Y directions, sufficient for us to identify the same biological microstructures as in the first imaging session. More precise microregistration could be achieved with further fine-tuning by adjusting the translational stage step motor (Figure 1f,g). In Figure 1f, the relative positions between the green and red dots demonstrated the precision of microregistrations. The red dot represents the ROI center point of the baseline experiment, and the green dots represent the ROI center points of the follow-up experiments.

### 3.2. Imaging the Dynamics of Skin Cellular Responses to UVB Exposure

UVB exposure is one of the major risk factors for inducing skin cancer. Previous studies have utilized multiphoton laser tomography to study UVB effects on skin in vivo [12,13]. Incorporating the microregistration method into a multimodal multiphoton microscopy system, we were able to investigate the dynamics of cells and tissue microstructures of human skin in vivo for two weeks following an acute UVB exposure. Supplemental Figure S3 shows the microscopy system interfaced with the surface marker and the imaging skin site. The system provides co-registered, simultaneous reflectance confocal microscopy (RCM) imaging, two-photon (excitation) fluorescence (TPF) microscopy imaging, and second harmonic generation (SHG) microscopy imaging. Two different excitation wavelengths were selected for acquiring these images: 735 nm and 810 nm. The three imaging modalities provide complimentary morphological and biochemical information [10]. The 735 nm fs laser-excited TPF imaging is ideal for visualizing epidermal cells [14] since the major cellular fluorophores NAD(P)H and melanin become nicely visible. The SHG signal excited at this wavelength is relatively very weak. We observed that under laser excitation of 810 nm, the TPF intensity of NAD(P)H is much less compared to 735 nm excitation. Therefore, the TPF intensity of melanin is more apparent. Also, SHG signals excited at this wavelength are very strong, which is ideal for imaging dermal collagen structures [14]. 

The study design is shown in Supplemental Figure S4. The skin site was first imaged at baseline before UVB exposure, and then imaged again at 1 h, 24 h, 3 days, 7 days, and two weeks after UVB exposure. The study protocol was approved by the Clinical Research Ethics Board of the University of British Columbia (certificate number: H16-00355) and consent was obtained from each volunteer subject before the experiment. The UVB exposure dose was 2 MED (minimum erythema dose).

We also acquired z-stacks of confocal (RCM) and multiphoton (TPF and SHG) images (see Supplemental Videos S1–S5). Figure 2a shows the horizontally scanned multimodal images of the same ROI over two weeks before and after UVB exposure at different skin layers: stratum granulosum (SG), stratum spinosum (SS), and stratum basale (SB) (green color codes for 735 nm fs laser-excited TPF signals, while magenta is for 810 nm fs laser-excited TPF). Melanin has strong TPF signals under both 735 nm and 810 nm excitation and thus appears white (green plus magenta). Figure 2b shows vertically scanned images corresponding to the same time points as in Figure 2a. Here, cyan color is coded for the SHG signal under 810 nm excitation, and red is for RCM signals at the same excitation wavelength. Figure 2c shows multimodal en face images of the dermis before, 1 h, and 24 h after UVB exposure. SHG (cyan) and standard deviation (STD) of the RCM intensity (red) at each pixel were calculated from ten sequential image frames to highlight blood flow in vessels. The STD analysis method was described in our previous publications [11]. 

For the SG layer, there was no obvious difference immediately before and after UVB exposure. At the SB layer, the TPF signal was mainly attributed to melanin in the cytoplasm. The presumed melanin signal became prominent by overlaying 810 nm and 735 nm-excited TPF images. In Figure 2a, the presumed melanin started to migrate from deeper layers to the SG layer from day 7 onwards, and became prominent after two weeks (white dots in Figure 2a SG layer). Cellular dynamics within the SS layer were different from those of the SG layer. Cell density increased, while cell size decreased within 24 h, which may indicate UVB-induced cell proliferation. One prominent feature of this layer was the presumed melanin that appeared on day 3 (Figure 2a SS layer). The cells in the SB layer are much smaller than those in the SG and SS layers. Due to the existence of melanocytes in this layer, the response of cells to UVB exposure was quite different from the SG and SS layers. Presumed melanin signals were also increased from day 3 all the way to two weeks, corresponding to delayed pigment darkening, and were composed of nests and clusters of pigmented cells [15]. The dermal also showed responses to UVB-induced injury. Before UVB exposure, capillary blood flow was barely perceptible, but then right after UVB, blood flow velocity immediately increased. This increased blood flow velocity persisted for 3 days, and then decreased at 1 and 2 weeks (Figure 2c).

### 3.3. Presumed Melanin Quantification

Melanin exhibits two-photon fluorescence, allowing for its in vivo assessment, and it is comparable to histology studies [16,17]. It is known that during the tanning process, the amount of melanin may increases [18,19,20]. However, how melanin concentration changes in response to low-dose UV remains controversial [21,22,23]. Besides the concentration, the distribution of melanin may also contribute to the process. A recent study using histology has shown that melanin from the lower layer migrates upwards to the middle layer of the skin post-UV radiation [18]. In this study, we demonstrated presumed melanin TPF signal intensity at each depth over time, allowing visualization of the upward transit of presumed melanin within the epidermis for the first time ever (Figure 2e). We found five out of seven volunteers have shown a similar trend. We imaged the skin using two-photon fluorescence (TPF) at two excitation wavelengths: 735 nm and 810 nm. Using 735 nm excitation, there are other fluorophores (keratin, NAD(P)H) interfering with melanin. However, at 810 nm excitation, the keratin and NAD(P)H fluorescence signal intensity becomes far less observable. Therefore, we overlaid 730 nm and 810 nm images to better visualize the presumed melanin distribution (Figure 2a, the white-colored areas). To quantify the presumed melanin intensity, only the 810 nm images were used for calculations. The SC layer was excluded in this analysis because keratin shows strong fluorescence intensities. From baseline to 2 weeks, the number of cells appearing white increased over time, especially at the stratum granulosum (SG) and spinosum (SS) layers. We further integrated the white signal within the imaging stack at depths from the top of the SG up to the dermal-epidermal junction (DEJ). Volumetrically, the calculated presumed melanin intensity increased up to 1.6-fold within 2 weeks post-UVB exposure (Figure 2e, insertion bar graph) as a delayed pigmentation response. Delayed pigment darkening or delayed tanning is a phenomenon induced by sunlight exposure, as a result of increased epidermal melanin formation. From our data (see Supplemental Figure S7d), the increasing trend of the presumed melanin TPF signal intensity was inconsistent with previous multiphoton microscopy measurements [13].

### 3.4. Tissue Architecture

We investigated the cell density changes with time. The total number of cells was counted for the SS and SG layers over time and is depicted in Figure 2d and Supplemental Figure S7c. The number of cells within the SS layer increased with time, reaching a maximum at 24 h post-UVB exposure and then dropping back at 3 d. The increasing trend of cellular density is presumed to be related to proliferation, which has been known as a self-repair mechanism. Our result is in accordance with previous reports using the immunohistochemistry staining method [24]. Hyperkeratosis has been confirmed to be most prominent during 24–48 h, decreasing after 72 h [24]. At the unexposed control site, the cell density was not changed.

### 3.5. Epidermal Thickness

Exposure to UV light leads to an increase in epidermal thickness, which helps to protect the skin against UV penetration [25]. The ET depends on the anatomical location and other factors such as age, gender, skin type, pigmentation, etc. Standard measurements of ET are performed with the histology measurement, with the averaged ET of the dorsal forearm being around 55 μm [26]. For in vivo ET measurement by multiphoton microscopy, vertical section images were usually indirectly generated by reconstructing the horizontal scanned images [27,28]. It is said that the gold standard cryohistology measured values might be different from the in vivo measurements since the former were determined in only five predefined locations within each vertical sectioned image. The strongly undulating nature of the DEJ makes it easy to miss the big picture of the epidermal anatomy [29]. In our study, we measured the epidermal thickness non-invasively by direct vertical scanned multiphoton skin images as shown in Figure 2b. The skin site’s (upper inner arm) average ET is 40.4 μm; the upper inner arm tends to have a thinner epidermis than the dorsal arm. Our measurement is inconsistent with other studies [12,13,16]. To determine the dermal-epidermal junction (DEJ), both the SHG image of collagen (cyan color) and the RCM image (red color) were used. Epidermal thickness increased as much as 20% above baseline between 24 h and one week, then decreased to 10% above baseline at two weeks. The averaged ET increased from 33.24 μm (baseline) up to 40.41 μm (3 days), then decreased to 38.53 two weeks later (see Supplemental Figure S6a). The thickness of the stratum corneum (SC) layer gradually increased from 12.0 μm up to 16.62 μm post-2 weeks exposure (see Supplemental Figure S7b). As a comparison at the control site where there was no UVB exposure, the thickness of the stratum corneum and epidermis was not changed (Figure 2f).

## 4. Discussion

The effects of UVB on human skin have been investigated, generating varying results and conclusions. The contradictory results may be affected by many factors, such as the dose of UVB, single or multiple dose administration, volunteer skin type, body site, etc. In a previous UVB study [12], the UVB dose used was 0.24 J/cm^2^. This high dose may induce apoptosis or even blistering; thus, the trends of spongiosis, pigmentation, and epidermal thickness are much more prominent. In another study [13], the UVB dose was 0.55–6.15 J/cm^2^ in total, but was applied each day stepwise with a daily dose in the range of 0.1–0.55 J/cm^2^ for 4–14 treatment days. In this case, the results may indicate photoadaptation of the skin, which is quite different from the effect of single-dose irradiation. Another factor that needs to be considered is biological variation; the same UVB dose may induce different biological effects on subjects with different skin types. For example, it needs a higher UVB dose to induce the same erythema level for a darker skin volunteer than for a lighter skin volunteer. Therefore, in our study, the UVB dose was designed to be 2 MED for all volunteers; thus, the dose value for each volunteer was different, ranging from 0.029 to 0.071 J/cm^2^, which is also much lower than the 0.24 J/cm^2^ dose used in the above study [12]. So, it was expected that our conclusion would be different from previous studies. The uniqueness of our study is that we developed a precise relocalization method to track the same cells over 2 weeks. The significant difference is that our method eliminated the site variability when taking images at different time points/sessions. For other multiphoton microscopy studies, multiple sites were randomly selected at each time point, and statistical analysis was performed to average out the site variability in order to get a meaningful average trend of variations. Our method eliminated the need for that kind of statistical approach since we were measuring the same microsite/same cells. We reduced the number of study sites or subjects required significantly.

The microcregistration method in conjunction with skin imaging technologies may provide new avenues for skin research and patient care. We applied this method in normal human skin for cell tracking for over two weeks [30]. And we recently extended the time up to three weeks by applying a secondary temporary tattoo. A recent study has shown that individual cell motion in skin microvessels is predictive of hematologic cancer relapse after stem cell transplant [31]. In their study, longitudinal monitoring was achieved using hair follicles and hair as landmarks [32]. However, easy-to-track landmarks cannot always be found in given experimental conditions. For example, if the imaged skin site has no visible hair, or hair needs to be removed, it is more convenient to use our temporary tattoo as a landmark for tracking. In our study, the upper inner arm site has less obvious hair follicles, and we found our temporary tattoo-based microcregistration method is very effective.

## 5. Conclusions

In conclusion, we developed a method to achieve precise serial microregistration for in vivo microscopy of human skin to track skin cells and other microstructures at separate imaging sessions over a 2-week period following acute UVB exposure. To our knowledge, this is the first study to directly monitor UVB-induced cellular evolution at the same microlocation over a 2-week period non-invasively in human skin. Cellular responses to UVB exposure, including cell proliferation, melanin upward migration, increase in epidermal thickness, delayed pigment darkening, and increased blood flow velocity in the dermal layer were observed. This technique opens the possibility for in vivo, non-invasive, and label-free longitudinal studies in humans, such as monitoring cancer cell metastasis, stem cell evolution, acute and chronic inflammation, wound healing, or drug delivery. It could be used with all kinds of microscopy modalities, existing or under development.

## Data Availability

All data needed to evaluate the conclusions in this paper are present in this paper and the Supplementary Materials. Any data reported will be shared by the lead contact upon request. Any additional information required to reanalyze the data reported in this paper is available from the lead contact upon request.

## Supplementary Materials

The following supporting information can be downloaded at: https://www.mdpi.com/article/10.3390/cells13131158/s1, Table S1: Steps of coordinate transformation; Supplemental Note S1: Procedure of applying the surface marker; Supplemental Note S2: Method to relocalize to the same cells/microstructure; Figure S1: Prediction algorithm for microregistration; Figure S2: Detailed experimental procedure of microregistration; Figure S3: The multimodal microscopy system; Figure S4: Experimental design; Figure S5: Delayed pigmentation analysis; Figure S6: Detailed 3D illustration of the ring adaptor; Figure S7: Changes in epidermal thickness, stratum corneum thickness, cell density, and melanin intensity; Supplementary videos S1–S3: z-stacks TPF, SHG, RCM images acquired under 810 nm fs laser excitation; Supplementary videos S4–S5: z-stacks TPF and RCM images acquired under 735 nm fs laser excitation.

## References

[1] Keller P.J., Schmidt A.D., Santella A., Khairy K., Bao Z., Wittbrodt J., Stelzer E.H.K. (2010). Fast, High-Contrast Imaging of Animal Development with Scanned Light Sheet–Based Structured-Illumination Microscopy. Nat. Methods.

[2] Chen B.-C., Legant W.R., Wang K., Shao L., Milkie D.E., Davidson M.W., Janetopoulos C., Wu X.S., Hammer J.A., Liu Z. (2014). Lattice Light-Sheet Microscopy: Imaging Molecules to Embryos at High Spatiotemporal Resolution. Science.

[3] Schroeder T. (2008). Imaging Stem-Cell-Driven Regeneration in Mammals. Nature.

[4] Skylaki S., Hilsenbeck O., Schroeder T. (2016). Challenges in Long-Term Imaging and Quantification of Single-Cell Dynamics. Nat. Biotechnol..

[5] Zipfel W.R., Williams R.M., Webb W.W. (2003). Nonlinear Magic: Multiphoton Microscopy in the Biosciences. Nat. Biotechnol..

[6] Helmchen F., Denk W. (2005). Deep Tissue Two-Photon Microscopy. Nat. Methods.

[7] Schroeder T. (2011). Long-Term Single-Cell Imaging of Mammalian Stem Cells. Nat. Methods.

[8] Entenberg D., Voiculescu S., Guo P., Borriello L., Wang Y., Karagiannis G.S., Jones J., Baccay F., Oktay M., Condeelis J. (2018). A Permanent Window for the Murine Lung Enables High-Resolution Imaging of Cancer Metastasis. Nat. Methods.

[9] Jung Y., Tam J., Ray Jalian H., Rox Anderson R., Evans C.L. (2015). Longitudinal, 3D In Vivo Imaging of Sebaceous Glands by Coherent Anti-Stokes Raman Scattering Microscopy: Normal Function and Response to Cryotherapy. J. Investig. Dermatol..

[10] Wang H., Lee A.M.D., Frehlick Z., Lui H., McLean D.I., Tang S., Zeng H. (2013). Perfectly Registered Multiphoton and Reflectance Confocal Video Rate Imaging of in Vivo Human Skin. J. Biophotonics.

[11] Huang Y., Wu Z., Lui H., Zhao J., Xie S., Zeng H. (2019). Precise Closure of Single Blood Vessels via Multiphoton Absorption–Based Photothermolysis. Sci. Adv..

[12] Koehler M.J., Kellner K., Hipler U.-C., Kaatz M. (2015). Acute UVB-induced Epidermal Changes Assessed by Multiphoton Laser Tomography. Skin. Res. Technol..

[13] Koehler M.J., Kellner K., Kaatz M., Hipler U.-C. (2016). Epidermal Changes during UVB Phototherapy Assessed by Multiphoton Laser Tomography. Skin. Res. Technol..

[14] Breunig H.G., Studier H., König K. (2010). Multiphoton Excitation Characteristics of Cellular Fluorophores of Human Skin in Vivo. Opt. Express.

[15] Majdzadeh A., Lee A.M.D., Wang H., Lui H., McLean D.I., Crawford R.I., Zloty D., Zeng H. (2015). Real-time Visualization of Melanin Granules in Normal Human Skin Using Combined Multiphoton and Reflectance Confocal Microscopy. Photoderm. Photoimm. Photomed..

[16] Ait El Madani H., Tancrède-Bohin E., Bensussan A., Colonna A., Dupuy A., Bagot M., Pena A.-M. (2012). In Vivo Multiphoton Imaging of Human Skin: Assessment of Topical Corticosteroid-Induced Epidermis Atrophy and Depigmentation. J. Biomed. Opt..

[17] Saager R.B., Balu M., Crosignani V., Sharif A., Durkin A.J., Kelly K.M., Tromberg B.J. (2015). In Vivo Measurements of Cutaneous Melanin across Spatial Scales: Using Multiphoton Microscopy and Spatial Frequency Domain Spectroscopy. J. Biomed. Opt..

[18] Tadokoro T., Yamaguchi Y., Batzer J., Coelho S.G., Zmudzka B.Z., Miller S.A., Wolber R., Beer J.Z., Hearing V.J. (2005). Mechanisms of Skin Tanning in Different Racial/Ethnic Groups in Response to Ultraviolet Radiation. J. Investig. Dermatol..

[19] Hennessy A., Oh C., Diffey B., Wakamatsu K., Ito S., Rees J. (2005). Eumelanin and Pheomelanin Concentrations in Human Epidermis before and after UVB Irradiation. Pigment. Cell Res..

[20] Wolber R., Schlenz K., Wakamatsu K., Smuda C., Nakanishi Y., Hearing V.J., Ito S. (2008). Pigmentation Effects of Solar-simulated Radiation as Compared with UVA and UVB Radiation. Pigment. Cell Melanoma Res..

[21] Majno G., Palade G. (1961). STUDIES ON INFLAMMATION: I. The Effect of Histamine and Serotonin on Vascular Permeability: An Electron Microscopic Study. J. Cell Biol..

[22] Willis I., Kligman A., Epstein J. (1972). Effects of Long Ultraviolet Rays on Human Skin: Photoprotective or Photoaugmentative?. J. Investig. Dermatol..

[23] Kaidbey K.H., Kligman A.M. (1975). Further Studies of Photoaugmentation in Humans: Phototoxic Reactions. J. Investig. Dermatol..

[24] Lee J.H., An H.T., Chung J.H., Kim K.H., Eun H.C., Cho K.H. (2002). Acute Effects of UVB Radiation on the Proliferation and Differentiation of Keratinocytes. Photoderm. Photoimm. Photomed..

[25] De Winter S., Pavel S., Vink A.A., Roza L. (2001). Solar-Simulated Skin Adaptation and Its Effect on Subsequent UV-Induced Epidermal DNA Damage. J. Investig. Dermatol..

[26] Sandby-Møller J., Poulsen T., Wulf H.C. (2003). Epidermal Thickness at Different Body Sites: Relationship to Age, Gender, Pigmentation, Blood Content, Skin Type and Smoking Habits. Acta Dermato-Venereol..

[27] Balu M., Kelly K.M., Zachary C.B., Harris R.M., Krasieva T.B., König K., Durkin A.J., Tromberg B.J. (2014). Distinguishing between Benign and Malignant Melanocytic Nevi by In Vivo Multiphoton Microscopy. Cancer Res..

[28] Decencière E., Tancrède-Bohin E., Dokládal P., Koudoro S., Pena A., Baldeweck T. (2013). Automatic 3D Segmentation of Multiphoton Images: A Key Step for the Quantification of Human Skin. Skin. Res. Technol..

[29] Verdel N., Lentsch G., Balu M., Tromberg B.J., Majaron B. (2018). Noninvasive Assessment of Skin Structure by Combined Photothermal Radiometry and Optical Spectroscopy: Coregistration with Multiphoton Microscopy. Appl. Opt..

[30] Tian G., Lui H., Zhao J., Wu Z., Kalia S., Richer V., Seo I., Ouyang H., Zeng H. Tracking Cellular Dynamics of Human Skin Responses to UV Exposure Using in Vivo Multimodal Microscopy (Conference Presentation). Proceedings of the Photonics in Dermatology and Plastic Surgery.

[31] Saknite I., Patrinely J.R., Zhao Z., Chen H., Beeghly-Fadiel A., Kim T.K., Jagasia M., Byrne M., Tkaczyk E.R. (2022). Association of Leukocyte Adhesion and Rolling in Skin with Patient Outcomes After Hematopoietic Cell Transplantation Using Noninvasive Reflectance Confocal Videomicroscopy. JAMA Dermatol..

[32] Saknite I., Byrne M., Jagasia M., Tkaczyk E.R. Confocal Video Microscopy Reveals Altered Leukocyte-Endothelial Interactions in Skin Preceding Acute Graft-versus-Host Disease (Conference Presentation). Proceedings of the Photonics in Dermatology and Plastic Surgery.

