# Supplementary material for: Precise Serial Microregistration Enables Quantitative Microscopy Imaging Tracking of Human Skin Cells In Vivo
Source: Cells. 2024 Jul 7;13(13):1158. doi: 10.3390/cells13131158 (PMC11240548; doi:10.3390/cells13131158)
Supplement: Supplementary file 1 [file cells-13-01158-s001.zip › Supplementary Materials/YT et al Zeng Suppl Mat Submision.pdf]

Supplementary Materials

Precise serial microregistration enables quantitative microscopy imaging tracking of human skin cells *in vivo*

Yunxian Tian[1, 2], Zhenguo Wu[1, 2] Harvey Lui[1, 2], Jianhua Zhao[1, 2], Sunil Kalia[2], InSeok Seo[3], Hao Ou-Yang[3], Haishan Zeng[1, 2, *]

1 Imaging Unit, Integrative Oncology Department, BC Cancer Research Centre, Vancouver, BC, Canada;
2 Photomedicine Institute, Department of Dermatology and Skin Science, University of British Columbia and Vancouver Coastal Health Research Institute, Vancouver, BC, Canada;
3 Johnson and Johnson Consumer Inc, Skillman, NJ, USA;
* Correspondence: hzeng@bccrc.ca; Tel.: +1 604 675 8083

Supplementary Video Captions

Supplementary videos 1. z-stacks TPF images acquired under 810 nm fs laser excitation.

Supplementary videos 2. z-stacks SHG images acquired under 810 nm fs laser excitation.

Supplementary videos 3. z-stacks RCM images acquired under 810 nm fs laser excitation.

Supplementary videos 4. z-stacks TPF images acquired under 735 nm fs laser excitation.

Supplementary videos 5. z-stacks RCM images acquired under 735 nm fs laser excitation.

Supplemental Table S1

Steps of coordinate transformation

	T0: Fist time visit	T1: Follow up visit
Known coordinates	$\binom{x_1}{y_1}$ $\binom{x_2}{y_2}$ $\binom{x_3}{y_3}$	$\binom{x'_1}{y'_1}$ $\binom{x'_2}{y'_2}$
1 center points	$\binom{Centx_{t0}}{Centy_{t0}} = \frac{1}{2}\left[\binom{x_1}{y_1} + \binom{x_2}{y_2}\right]$	$\binom{Centx_{t1}}{Centy_{t1}} = \frac{1}{2}\left[\binom{x'_1}{y'_1} + \binom{x'_2}{y'_2}\right]$
2 Translation	$T_{t0} = \binom{Centx_{t0}}{Centy_{t0}}$	$T_{t1} = \binom{Centx_{t1}}{Centy_{t1}}$
3 Rotation	$\binom{x_{1n}}{y_{1n}} = \binom{x_1}{y_1} - T_{t0}$ $\theta = arctan\,(x_{1n}/y_{1n})$ $R_{t0} = \begin{pmatrix} cos\theta & -sin\theta \\ sin\theta & cos\theta \end{pmatrix}$	$\binom{x'_{1n}}{y'_{1n}} = \binom{x'_1}{y'_1} - T_{t1}$ $\varphi = arctan\,(x'_{1n}/y'_{1n})$ $R_{t1} = \begin{pmatrix} cos\varphi & -sin\varphi \\ sin\varphi & cos\varphi \end{pmatrix}$
4 Scale	$L_{t0} = \sqrt[2]{{x_{1n}}^2 + {y_{1n}}^2}$	$L_{t1} = \sqrt[2]{{x'_{1n}}^2 + {y'_{1n}}^2}$ $Sy = L_{t1}/L_{t0}$ $S_{t1} = \binom{1}{Sy}$
5 Transformation	$\binom{x_{3t0}}{y_{3t0}} = R_{t0} * \left[\binom{x_3}{y_3} - T_{t0}\right]$	$S_{t1} * \binom{x_{3t0}}{y_{3t0}} = R_{t1} * \left[\binom{x_{3t1}}{y_{3t1}} - T_{t1}\right]$
6 Solution	$\binom{x_{3t1}}{y_{3t1}} = S_{t1} * {}^{R_{t0}}/_{R_{t1}} * \left[\binom{x_3}{y_3} - T_{t0}\right] + T_{t1}$	

Supplemental Note 1

Procedure of applying the surface marker:

1. Make sure the skin is clean, dry and free of any oil or the surface marker will not adhere. If the subject's skin has lotion or any other cosmetic products on, use a little soap and water or rubbing alcohol pad to clean the area.
2. Cut out the surface marker, and remove the clear sheet.
3. Before position the surface marker, make sure the skin does not twist or stretch.
4. Place the surface marker face down on the skin and hold a wet cloth against it for about 30 seconds.
5. Peel the paper aside then pat gently with the wet cloth.
6. Do not twist or stretch skin until the surface marker has had time to set (at least 5 minutes).

Supplemental Note 2

Method to relocalize to the same cells/microstructure

Baseline

Step 0: Apply surface marker to the skin site for imaging according to procedures in Supplementary Note 1.

Step 1: Place one droplet of water on the skin site to be imaged. Wait about 1 minute until the skin is hydrated. Gently absorb the excessive water using napkin. Prepare the imaging ring adaptor. The ring adaptor is a customized magnetic part. When doing imaging, the bottom side of the ring adaptor is affixed onto the coverslip and the skin using double-sided adhesive film, while the other side is attached to the imaging window by magnetic attraction. This adaptor is used to minimize the involuntary movement between patient skin and the objective. Place a ring shaped double-sided adhesive film on the back of the ring adaptor, align the round shaped cover slip to the center of the adaptor, and gently press down. Place a second ring shaped double sided adhesive film on the cover slip. Align the ring adaptor to the center of the surface marker and gently press against the skin for about 5 seconds. The ring adaptor mated magnetically to the imaging holder, which is connected to a motorized driven, micrometer-actuated 3-axis translation stage for controlling the imaging location and focal plane position inside the skin. Therefore the volunteer' skin is attached to the imaging window by the ring adaptor. (Supplimentary Fig.S6). When doing skin imaging, patient is asked to lie on a bed, his/her arm is resting on a cushioned stool to support the arm. The above method is efficiently avoided volunteer movement during imaging.

Step 2: Turn on the white LED light and the white light CCD camera. Adjust the z stage until the whole surface marker is within the white light (WL) imaging field of view (FOV). Gently rotate the ring adaptor until the surface marker looks aligned vertically in the FOV (coarse adjustment). The diameter of the ring adaptor is much larger than the surface marker, therefore the skin was not likely to be stretched since the surrounding was fixed by the double sided tape. The tuning process has negligible effect on the skin structure we imaged as long as: 1) the rotation angle is small (only few degrees), 2) the arm rotates with the ring as much as possible, 3) the remaining skin movement is minimum and also the whole thickness of the skin is moving together since the ring is attached to the skin. In the white light imaging software panel, manually draw a straight line, from the ref point 1 to point 2. Fine adjust the orientation until the straight line is vertically aligned. The fine adjustment was done by rotating the wings of the ring adaptor carefully while looking at the white light imaging. The adjustment was finished until the surface marker line was aligned with the vertical line in the white light imaging. Use the software to capture an image. Fix the imaging ring adaptor and imaging holder using tight screws. Place water between the objective and imaging ring adaptor. Stage travels until reference point 1 present in the center of FOV. Turn off white LED light.

Step 3: Before turning on RCM mode, check the incident laser power to be no larger than 10 mW, and tune down the APD detector gain. This step is to make sure the surface marker signal does not saturate the detectors since the surface marker is highly reflective. Adjust z stage to find the surface marker plane. If ref point 1 is off-centered, adjust xy stage until it is centered. Record the ref point 1 coordinates . Travel y stage until surface marker ref point 2 appears. Control xy stage to center the ref point 2. Record ref 2 coordinates Although rarely happen, if ref 2 is difficult to find, switch back to white light mode, center the ref point 2 under WL imaging. Switch back to RCM mode, fine tune the xy stage and record the coordinates.

Step 4: The center point is calculated by the prediction algorithm described in Supplementary Table S1 and Supplementary Figure S2. Stage travels to the center point. Browse the nearby site, and select ROI. Record coordinates.

Follow ups

Step 1,2 are the same as baseline.

Step 3: Follow the step3 in baseline to find the ref point 1 and ref 2 . Record the coordinates.

Step 4: Finding image site ROI is achieved by following the 5 sub steps shown below. Steps A&B is automatic relocalization. The mean time taken for steps A&B is about 2 minutes. Step C&D is manually fine tuning relocalization. These two steps only take a few seconds because we could see the images in real-time, and the adjustment of stage is easily controlled.

A. Calculate the predicted coordinates based on equation described in Supplementary Table S1 .

B. Stage travel to the position of the predicted coordinates.

C. Adjust z stage and visually examine the biological structure (such as vessel feature, DE junction) at all depth, and compare with baseline image stack.

D. If resemble, fine adjust xy stage until FOV is the same as that of baseline ROI. The experimental image site coordinates were recorded.

E. If D failed, increase incident laser power up to 40 mW and switch to SHG mode. Repeat the step C and D until the same FOV is found.

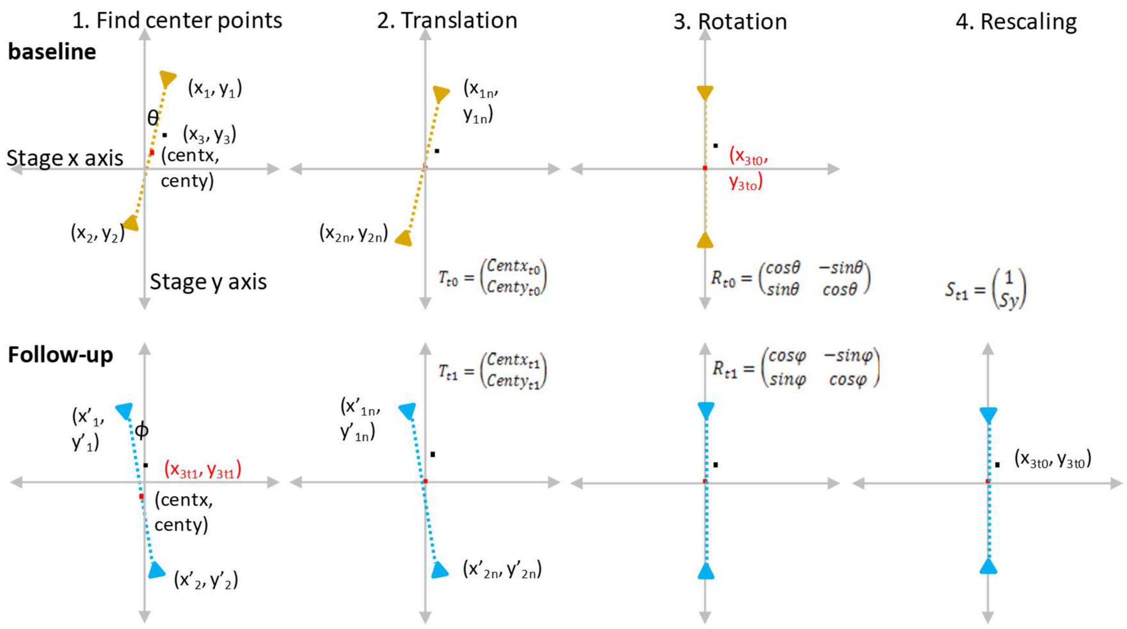

Figure S1. Prediction algorithm for microregistration. First row: coordinate transformation at baseline. Second row: coordinate transformation at a follow-up visit.

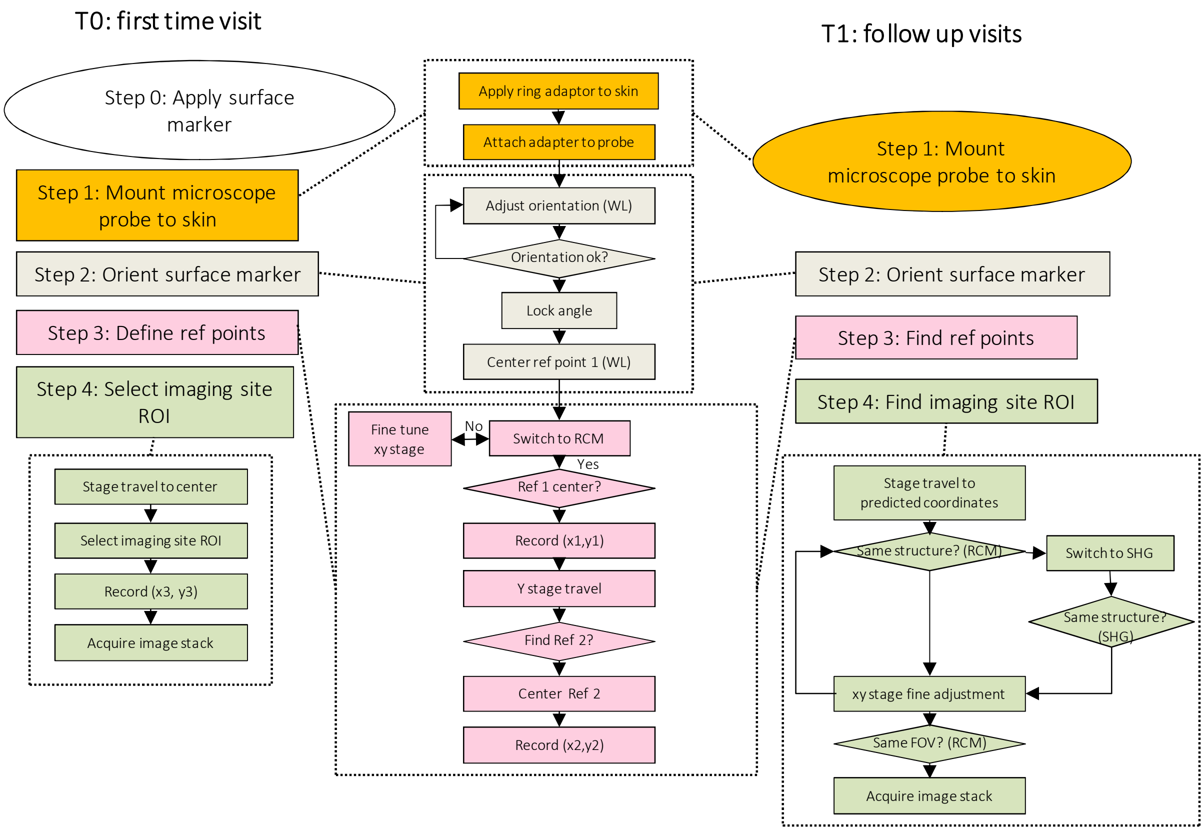

Figure S2. Detailed experimental procedure of microregistration.

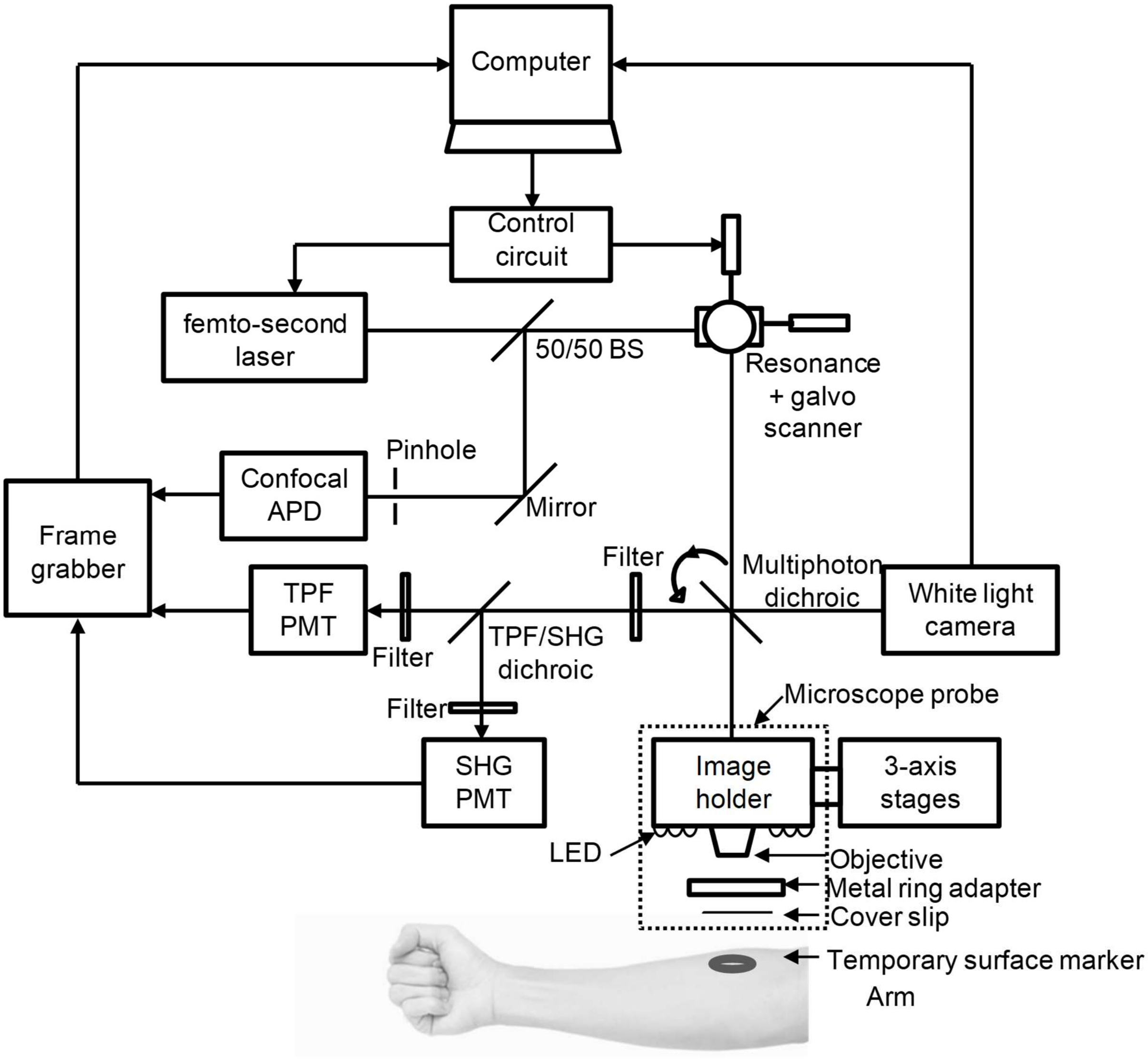

Figure S3. The multimodal microscopy system contains computer, control circuit, femto-second laser, resonance and galvo scanners, multiphoton dichroic mirror, imaging holder, objective lens, metal ring adaptor, cover slip, temporary surface marker, motorized micrometer-actuated 3-axis translation stage, white light camera, filters, SHG/TPF dichroic, SHG PMT, TPF PMT, frame grabber, confocal APD, and 50/50 BS (beam splitter). PMT – photomultiplier tube, APD – avalanche photodiode.

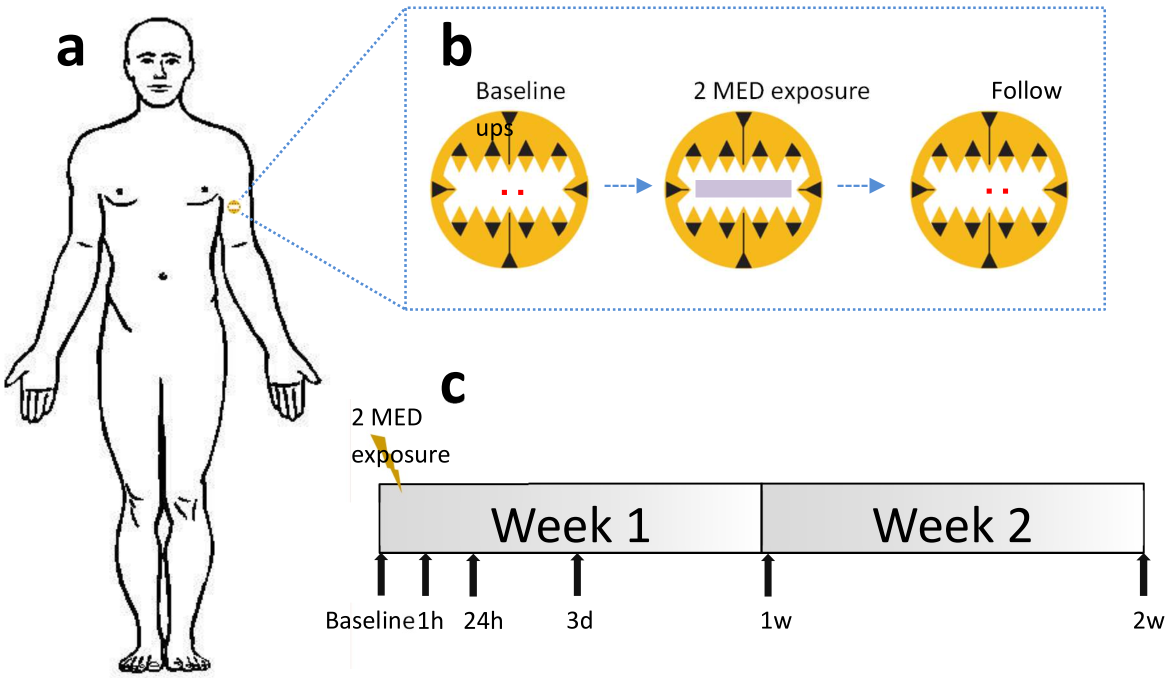

Figure S4. Experimental design. (a) The tested skin site of human volunteer is the upper inner arm. (b) At baseline, two region of interest (ROI) presented as red dots are selected and imaged. afterward, 2 MED UVB exposure is administrated at the central region of the open area of the surface marker. The same two ROI are localized and imaged at follow-up time points. (c) Study protocol summary: the duration of the experiment is 2 weeks with 6 time points (baseline, 1h, 24h, 3d, 1w, 2w) for measurements.

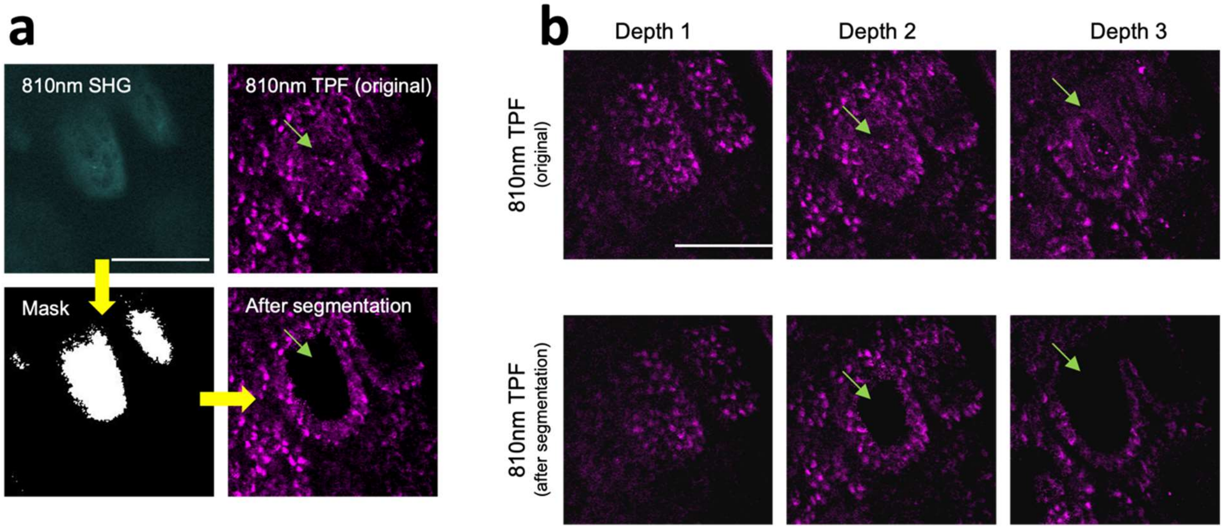

Figure S5. Delayed pigmentation analysis. (a) Top left: example of original 810 nm collagen SHG image, bottom left: binary mask was created by segmenting collagen signal, top right: original 810 nm TPF image, bottom right: 810 nm TPF image after masking the dermal signal. (b) 810 nm TPF original (first row) and segmented (second row) at three depths. Comparing to the original images, the elastin fibers and bright dots within the dermal region was excluded.

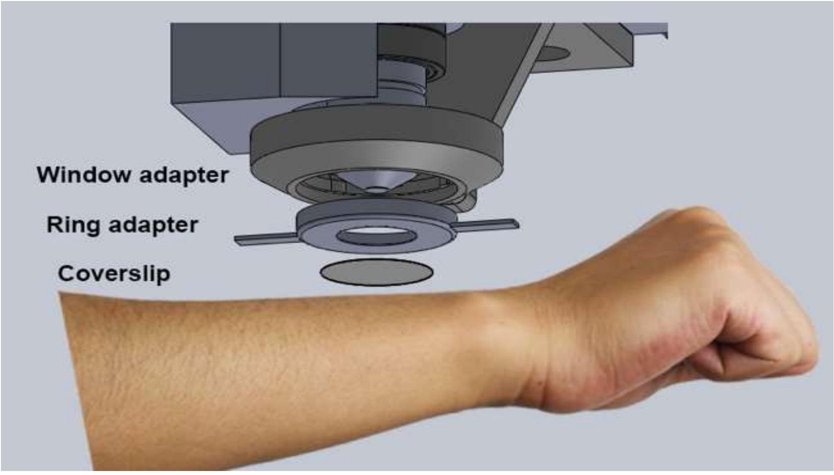

Figure S6. Detailed 3D illustration of the ring adaptor. The ring adaptor is a customized magnetic part. When doing imaging, the bottom side of the ring adaptor is affixed onto the coverslip and skin using double-sided adhesive film, while the other side is attached to the imaging window by magnetic attraction.

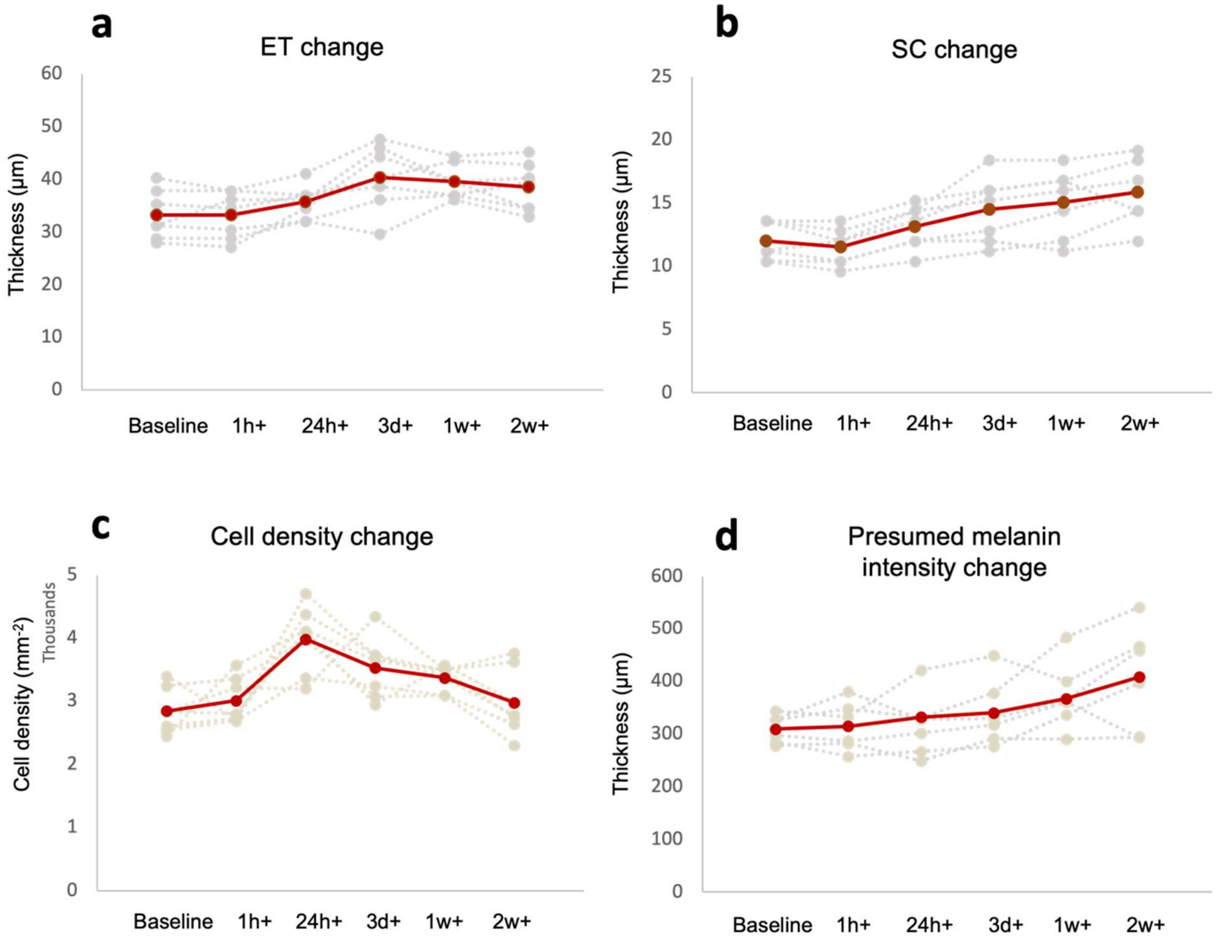

Figure S7 (a) Changes of total epidermal thickness (ET) after UVB exposure. The thickness of epidermis increases and then decreases between 24 hours and 2 weeks with a maximum at 3 days. (b) the stratum corneum (SC) thickness continues increasing over the 2 week period. (c) SS cell density changes after UVB exposure. Cell density in the SS layer increases following UVB exposure and reaches maximum at 24 hours, then settles down. (d) Total presumed melanin signal intensity changes over time. The intensity continues increasing over the 2 week period. Dotted line: individual volunteer. Red line: mean value of all volunteers' data.
